# Autophagy-Related Proteins’ Immunohistochemical Expression and Their Potential Role as Biomarkers in Thymic Epithelial Tumors

**DOI:** 10.3390/cancers18030357

**Published:** 2026-01-23

**Authors:** Christina Yfanti, Georgia Levidou, Vicky Lampropoulou, Stefania Kokkali, Georgios Mandrakis, Stavros P. Papadakos, Dimitra Rontogianni, Stamatios Theocharis

**Affiliations:** 1 First Department of Pathology, National and Kapodistrian University of Athens, 11527 Athens, Greece; christinayph99@gmail.com (C.Y.); stefikokkali@gmail.com (S.K.); giormandr@med.uoa.gr (G.M.); stavrospapadakos@gmail.com (S.P.P.); 2 Department of Pathology, Paracelsus Medical University, 90419 Nuremberg, Germany; georgia.levidou@klinikum-nuernberg.de; 3 Department of Microbiology, Medical School, National and Kapodistrian University of Athens, 11527 Athens, Greece; vlampro@med.uoa.gr; 4 Department of Pathology, Evangelismos General Hospital of Athens, 10676 Athens, Greece

**Keywords:** thymic epithelial tumors, autophagy, carcinogenesis, BECLIN, p62, LC3b, ATG3, prognostic significance, therapeutic targets

## Abstract

Background: Autophagy, a self-destructive cellular mechanism with a paradoxical nature, plays a part in both tumor suppression and induction by providing cancer cells with metabolic substrates, resulting in cell proliferation and survival. In this study, we aim to investigate the clinical significance of four autophagy pathway components (BECLIN, p62/, LC3b, ATG3) in pathogenetic mechanisms of thymic epithelial tumors (TETs) with possible prognostic importance. Methods: Immunohistochemistry was used to evaluate the cytoplasmic expression of BECLIN, p62, LC3b, and ATG3 in tumor cells of 99 TETs, and possible correlations with clinicopathological parameters were examined. Results: Higher BECLIN and p62 expression was associated with male gender (*p* = 0.027 and *p* = 0.014, respectively). B3 thymomas and thymic carcinomas (TCs) displayed higher p62 expression (*p* = 0.019), while LC3b expression was marginally higher in non-B3/TC TETs (*p* = 0.098). A positive correlation between higher BECLIN expression and advanced Masaoka–Koga stage was also observed (*p* = 0.009). ATG3 was not associated with any of the investigated clinicopathological parameters (*p* > 0.05). There was also no significant correlation between any of the four examined molecules and overall survival or relapse. Conclusions: Our findings indicate autophagy activation in B3/TC and advanced Masaoka–Koga stage cases. Further studies are needed to explore the role of these autophagy related proteins as potential biomarkers and therapeutic targets in TETs.

## Simple Summary

The present study investigated the role of autophagy, a cellular process, in thymic epithelial tumors (TETs) by analyzing four key proteins, BECLIN, p62, LC3b, and ATG3, using immunohistochemistry on 99 tumor samples. Higher BECLIN and p62 levels were linked to male patients and B3 thymomas/thymic carcinomas (TCs), while a positive correlation with advanced Masaoka–Koga stage was observed for BECLIN. Although LC3b showed a marginal increase in non-B3/TC TETs, ATG3 had no significant associations. While the study suggests that autophagy is active in more advanced or aggressive thymic cancers, these autophagy components did not significantly predict a patient’s overall survival or the likelihood of cancer relapse. Ultimately, the findings highlight autophagy as a potential area for future therapeutic targeting, though more research is needed to establish these proteins as reliable biomarkers.

## 1. Introduction

Thymic epithelial tumors (TETs) comprise a rare tumor entity in the anterior mediastinum displaying clinicopathological and molecular diversity and heterogeneity. They coexist with paraneoplastic autoimmune disorders, the main one of which is thymoma-associated myasthenia gravis (TAMG) [1,2], and according to WHO classification of tumors, these neoplasms are subdivided into distinct histological types, A, AB, B1, B2, B3 types of thymomas and thymic carcinomas (TCs) [3,4,5]. Thymomas of type A and AB are more often recognized by the GTF2I L424 missense mutation, HRAS mutations, and overexpression of the microRNA cluster on chromosome 19. On the other hand, thymomas type B and TCs display copy number alterations (CNAs) and more specifically gains of chromosome 7 and deletions on chromosome 16, respectively, as well as more frequent mutations of NRAS and TP53 genes [6,7,8,9,10,11]. TETs are characterized by the existence of low tumor mutation burden (TMB) [12,13], high expression of PD-L1 (Programmed Death-Ligand 1) in tumor cells [14,15,16,17,18,19,20,21], and epigenetic regulation of non-coding RNAs [22,23,24,25]. Nevertheless, the pathogenetic background in TETs still remains unknown.

Autophagy, first described in 1963 by a Belgian chemist, Christian de Duve, consists of a cellular self-destructive mechanism of degradation and recycling of cytotoxic products and damaged organelles and proteins, providing cells with new metabolic substrates and nutrients [26,27]. Autophagy is triggered by a wide range of stress signals, such as oxidative stress or a hypoxic and nutrient-deficient environment [28], and enables the maintenance of metabolic and cellular homeostasis by protecting organelles from harmful effects of reactive oxygen species (ROS). Simultaneously it regulates intracellular signaling pathways, resulting in cellular adaptation to stress conditions [29,30]. Several studies indicate the complex nature of autophagy, both participating in different cellular processes, for instance, differentiation, senescence, cell death, proliferation, immunomodulation, inflammation, organellar and molecular function, and genome remodeling [31,32], and demonstrating its prominent role in the pathogenesis of neurodegenerative, autoimmune and metabolic disorders, aging, and cancer [33].

Several studies have shed light on the paradoxical dual nature of autophagy in both promotion and inhibition of tumorigenesis, acting as a “double-edged sword”. The inductive or suppressing effect of autophagy on cancer depends on both tumor and host factors, such as the tumor type, stage of neoplastic disease, genetic profile, and tumor microenvironment [34]. While, in the early stages of tumor growth, autophagy degrades cytotoxic metabolic products and damaged molecules, suppressing tumorigenesis signals and exhibiting a cytoprotective role against cancer, in advanced tumor stages, autophagy enhances carcinogenesis due to its importance in providing cancer cells with nutrients and substrates, sustaining cancer growth in an environment of hypoxia and energy exhaustion based on glucose and glutamine deprivation [34,35]. Autophagy’s multidimensional function in tumor promotion is visible through its modulation of a variety of processes, such as angiogenesis [36], immune escape [37], metabolic stress [38,39], resistance to apoptosis [40], activation of cancer stem cells (CSCs) [41,42,43], promotion of epithelial–mesenchymal transition (EMT), infiltration and metastasis [30,44], epigenetic alterations [45,46,47], interaction with different types of cell death, such as ferroptosis, pyroptosis, and necroptosis [48,49,50], or even interaction with carcinogenic pathogens [51].

The expression of autophagy pathway components in TETs, as well as their pathogenetic function and their potential role as biomarkers and therapeutic targets, has not yet been described. Taking this into consideration, the present study aims to evaluate the immunohistochemical expression of four fundamental autophagy-related proteins (BECLIN, p62, LC3b, and ATG3), encoded by core autophagy genes (BECN1, SQSTM1, MAP1LC3B, and ATG3), and correlating expression patterns with clinicopathological parameters and patients’ survival in a cohort of 99 TETs, as they appear in a detailed way in Table 1.

## 2. Results

### 2.1. BECLIN Expression and Associations with Clinicopathological Features

**Figure 1:**
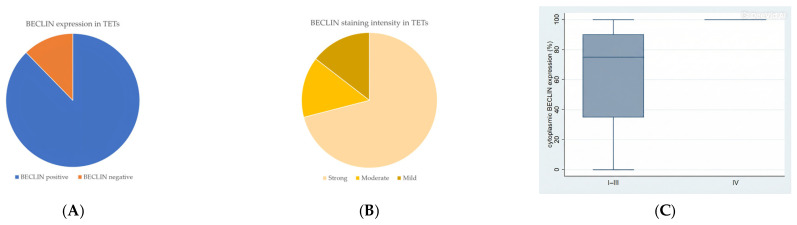
(**A**) Positivity rate of cytoplasmic BECLIN expression in TETs. (**B**) Distribution of BECLIN staining intensity in TETs. (**C**) Schematic representation of the associations between cytoplasmic BECLIN H-score expression and Masaoka–Koga stage (Mann–Whitney U test, Masaoka–Koga stage IIII versus IV, *p* = 0.009). Higher cytoplasmic BECLIN expression was observed in advanced Masaoka–Koga stage IV compared to Masaoka–Koga stages I–III. Horizontal lines represent medians, and whiskers show the full distribution excluding outliers. In stage IV, BECLIN expression shows minimal variability, with the box plot collapsing into a single line, indicating homogeneous expression levels across samples.

**Figure 2:**
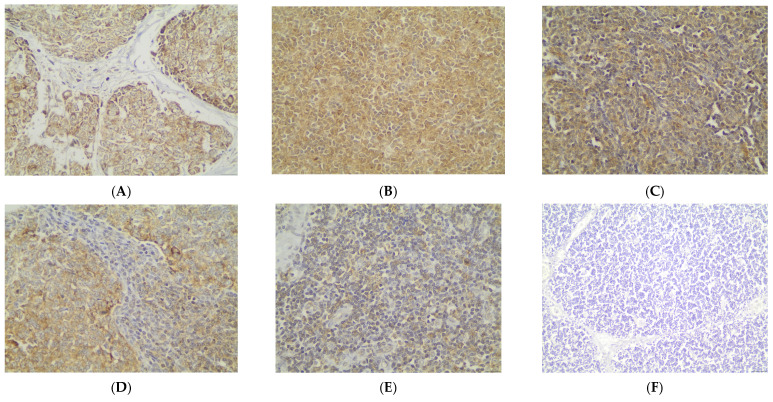
Immunohistochemical cytoplasmic BECLIN expression (**A**) in a thymic carcinoma (strong staining intensity), (**B**) in a type B3 thymoma (strong staining intensity), (**C**) in a type A thymoma (moderate staining intensity), (**D**) in a type AB thymoma (moderate staining intensity), (**E**) in a type B2 thymoma (mild staining intensity), and (**F**) in normal thymic tissue (×400).

Immunohistochemical cytoplasmic BECLIN expression was observed in the overwhelming majority of TETs in a percentage of 87.7% of the investigated cases, with a median value of expression of 80% (Table 2, Figure 1A). More specifically, 64 out of the 73 cases demonstrated BECLIN cytoplasmic immunopositivity (Figure 1A). Moreover, 88 of the immunoreactive cases displayed a strong BECLIN staining intensity (Figure 1B and Figure 2A,B), 18 a moderate one (Figure 1B and Figure 2C,D), and 18 a mild one (Figure 1B and Figure 2E). We can compare immunohistochemical cytoplasmic BECLIN positivity in TETs with the weakest to almost absent BECLIN expression in normal thymic tissue as depicted in Figure 2F.

Higher immunohistochemical cytoplasmic BECLIN expression was detected in males with TETs (Mann–Whitney U test, *p* = 0.027), with a median H-score of 87 and a range of 0–100 (Table 2). Furthermore, higher BECLIN immunopositivity was associated with an advanced Masaoka–Koga stage IV compared to Masaoka–Koga stages I–III (Mann–Whitney U test, Masaoka–Koga I–III versus IV, *p* = 0.009), with a median H-score of 100 and a range of 100–100 (Table 2, Figure 1C). There was no significant correlation between BECLIN immunoreactivity and WHO histological type of TETs (Mann–Whitney U test, non-B3/TC WHO histological type versus B3/TC). In addition, there was no significant association between BECLIN immunohistochemical expression and patients’ overall survival (<80% versus ≥80%, log rank), as well as the presence of relapse (Mann–Whitney U test) or the remaining clinicopathological parameters (Table 1).

### 2.2. p62 Expression and Associations with Clinicopathological Features

**Figure 3:**
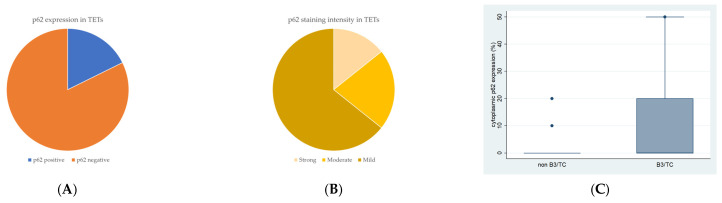
(**A**) Positivity rate of cytoplasmic p62 expression in TETs. (**B**) Distribution of p62 staining intensity in TETs. (**C**) Schematic representation of the associations between cytoplasmic p62 H-score expression and WHO histological type (Mann–Whitney U test, non-B3/TC WHO histological type versus B3/TC, *p* = 0.019). Higher cytoplasmic p62 expression was observed in type B3 thymomas and thymic carcinomas compared to non-B3/TC WHO histological types. Horizontal lines represent medians, and whiskers show the full distribution excluding outliers.

**Figure 4:**
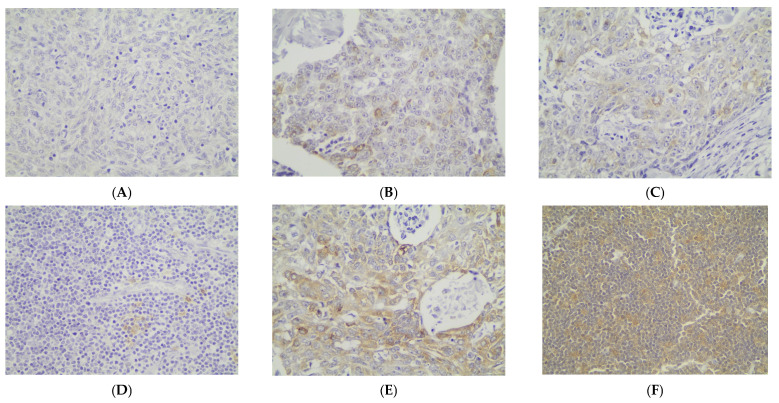
Immunohistochemical cytoplasmic p62 expression (**A**) in a type B2 thymoma (absence of staining), (**B**) in a type B3 thymoma (mild staining intensity), (**C**) in a thymic carcinoma (mild staining intensity), (**D**) in a type B1 thymoma (mild staining intensity), (**E**) in a type B3 thymoma (moderate staining intensity), and (**F**) in normal thymic tissue (×400).

Cytoplasmic p62 immunopositivity was encountered in only 14 out of the 79 cases of TETs, in a percentage of 17.7% of the examined cases (Table 2, Figure 3A), whereas 82.3% of the cases were not immunoreactive (Figure 3A and Figure 4A). Moreover, nine of the immunoreactive cases showed a mild p62 staining intensity (Figure 3B and Figure 4B–D), three a moderate one (Figure 3B and Figure 4E), and two a strong one (Figure 3B). We can compare immunohistochemical cytoplasmic p62 positivity in TETs with the weakest to almost absent p62 expression in normal thymic tissue as depicted in Figure 4F.

Higher immunohistochemical cytoplasmic p62 expression was present in males with TETs (Mann–Whitney U test, *p* = 0.014), with a median H-score of 0 and a range of 0–20 (Table 2). Furthermore, higher p62 immunopositivity was correlated with type B3 thymomas and thymic carcinomas compared to other WHO histological types of TETs (Mann–Whitney U test, non-B3/TC WHO histological type versus B3/TC, *p* = 0.019), with a median H-score of 90 and a range of 0–100 (Table 2, Figure 3C). There was no significant association between p62 immunoreactivity and Masaoka–Koga stage (Mann–Whitney U test, Masaoka–Koga I–II versus III–IV). In addition, there was no significant correlation between p62 immunohistochemical expression and patients’ overall survival (negativity versus positivity, log rank), as well as the presence of relapse (Mann–Whitney U test) or the remaining clinicopathological characteristics (Table 1).

### 2.3. LC3b Expression and Associations with Clinicopathological Features

**Figure 5:**
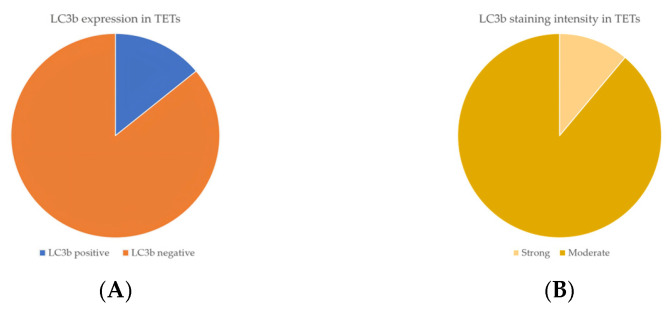
(**A**) Positivity rate of cytoplasmic LC3b expression in TETs. (**B**) Distribution of LC3b staining intensity in TETs.

**Figure 6:**
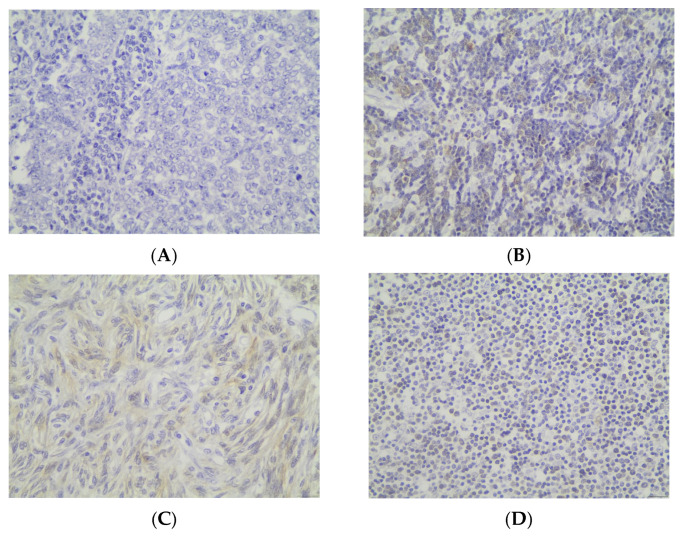
Immunohistochemical cytoplasmic LC3b expression (**A**) in a type B3 thymoma (absence of staining) (**B**) in a type B2 thymoma (moderate staining intensity) (**C**) in a type A thymoma (moderate staining intensity) (**D**) in normal thymic tissue (×400).

Cytoplasmic LC3b immunopositivity was observed in only 9 out of the 64 cases of TETs, in a percentage of 14.1% of the investigated cases (Table 2, Figure 5A), while 85.9% of the cases were not immunoreactive (Figure 5A and Figure 6A). Moreover, eight of the immunoreactive cases demonstrated a moderate LC3b staining intensity (Figure 5B and Figure 6B,C), and only one case showed a strong one (Figure 5B). We can compare immunohistochemical cytoplasmic LC3b positivity in TETs with the weakest to almost absent LC3b expression in normal thymic tissue as depicted in Figure 6D.

Immunohistochemical cytoplasmic LC3b expression was detected in non-B3-type thymomas and thymic carcinomas, but this association was of marginal significance (Fischer’s exact test, non-B3/TC WHO histological type versus B3/TC, *p* = 0.098) (Table 2). There was no significant correlation between LC3b immunopositivity and Masaoka–Koga stage (Fischer’s exact test, Masaoka–Koga I–II versus III–IV). Furthermore, there was no significant association between LC3b immunoreactivity and gender (Fischer’s exact test) or patients’ overall survival (negativity versus positivity, log rank), as well as the presence of relapse (Fischer’s exact test) or the remaining clinicopathological features (Table 1).

### 2.4. ATG3 Expression and Associations with Clinicopathological Features

**Figure 7:**
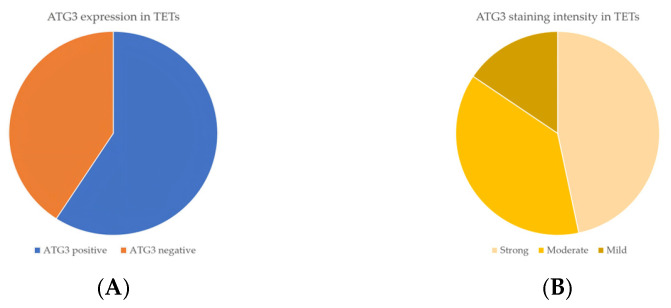
(**A**) Positivity rate of cytoplasmic ATG3 expression in TETs. (**B**) Distribution of ATG3 staining intensity in TETs.

**Figure 8:**
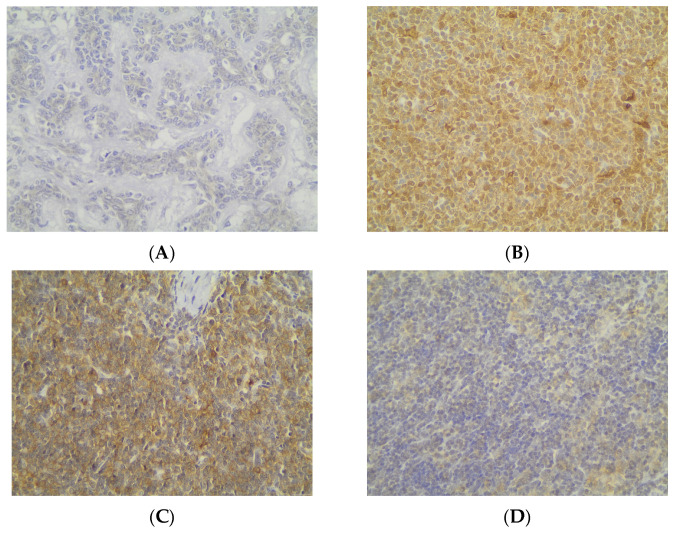
Immunohistochemical cytoplasmic ATG3 expression (**A**) in an MNT thymoma (absence of staining), (**B**) in a type B2 thymoma (moderate staining intensity), (**C**) in a type B2 thymoma (mild staining intensity), and (**D**) in normal thymic tissue (×400).

Immunohistochemical cytoplasmic ATG3 expression was encountered in a percentage of 59.2% of the examined cases, and more specifically, 45 out of the 76 cases displayed ATG3 cytoplasmic immunopositivity (Table 2, Figure 7A), whereas 40.8% of the cases were not immunoreactive (Figure 7A and Figure 8A). In addition, 21 of the immunoreactive cases showed a strong ATG3 staining intensity (Figure 7B), 17 a moderate one (Figure 7B and Figure 8B), and a mild one (Figure 7B and Figure 8C). We can compare immunohistochemical cytoplasmic AΤG3 positivity in TETs with the weakest to almost absent AΤG3 expression in normal thymic tissue as depicted in Figure 8D.

There was no significant correlation between immunohistochemical cytoplasmic ATG3 expression and WHO histological type of TETs (Mann–Whitney U test, non-B3/TC WHO histological type versus B3/TC) or Masaoka–Koga stage (Mann–Whitney U test, Masaoka–Koga I–II versus III–IV). Moreover, there was no significant association between ATG3 immunopositivity and gender (Mann–Whitney U test) or patients’ overall survival (negativity versus positivity, log rank), as well as the presence of relapse (Mann–Whitney U test) or the remaining clinicopathological parameters (Table 1).

### 2.5. Transcriptomic Profiling of Autophagy-Related Genes in TETs

**Figure 9:**
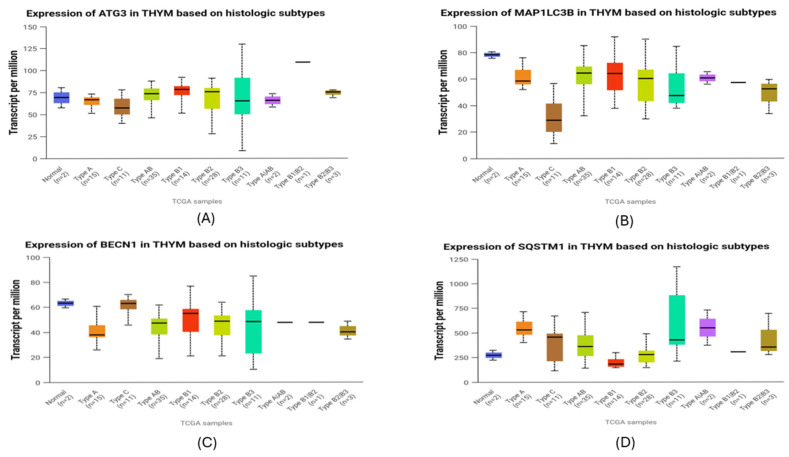
Transcriptomic expression of autophagy-related genes across histologic subtypes of thymic epithelial tumors (TCGA-THYM). (**A**) ATG3 expression patterns across subtypes. (**B**) MAP1LC3B (LC3B) transcript variation among histological subtypes. (**C**) BECN1 (Beclin-1) levels illustrating differences in autophagy-initiating activity. (**D**) SQSTM1 (p62) mRNA abundance showing subtype-dependent variation. Boxplots display transcript-per-million (TPM) values for each gene across normal thymus and major WHO histologic subtypes of TETs. Horizontal lines represent medians, and whiskers show the full distribution excluding outliers.

Transcriptomic analysis of autophagy-related genes in the TCGA-THYM cohort revealed pronounced subtype-specific differences (Figure 9). *ATG3* expression was markedly reduced in type C tumors, with significantly lower levels than in type AB (*p* = 0.0119), type B1 (*p* = 0.0206), and type B2 (*p* = 0.0120). *MAP1LC3B* followed a similar pattern, showing its lowest expression in type C and significantly decreased levels relative to type A (*p* = 0.00396), AB (*p* = 0.00026), B1 (*p* = 0.0024), and B2 (*p* = 0.0012). In contrast, *BECN1* was enriched in type C, where transcript abundance was significantly higher than in type A (*p* = 0.00778), AB (*p* = 0.00874), B1 (*p* = 0.0447), B2 (*p* = 0.0165), and B3 (*p* = 0.0266), indicating increased autophagy-initiating activity in this more aggressive subtype. *SQSTM1*, the gene encoding the p62 protein, showed substantial variation across histologic groups. Type A exhibited the highest SQSTM1 levels overall, significantly exceeding those of type C (*p* = 0.029), type AB (*p* = 0.0010), type B1 (*p* = 0.000002), and type B2 (*p* = 0.000198). Type B1 displayed significantly lower expression than type C (*p* = 0.012), AB (*p* = 0.00000011), B2 (*p* = 0.000918), and B3 (*p* = 0.0025). Additional differences included higher *SQSTM1* in type B3 compared with B2 (*p* = 0.013) and in type AB compared with B2 (*p* = 0.035). Despite this variability, *SQSTM1* remained the autophagy-related gene with the highest overall transcript abundance in TETs, indicating a broadly elevated baseline of p62 transcription. Correlation with immunohistochemistry showed varying gene–protein alignment. *BECN1* demonstrated strong concordance, as Beclin-1 protein increased in more advanced tumors in agreement with its transcript profile. LC3B also showed directional alignment, with both mRNA and protein levels being lowest in the most aggressive subtypes and higher in less aggressive thymomas. In contrast, p62 protein accumulation was greatest in aggressive tumors despite the variability in *SQSTM1* transcripts, reflecting only partial concordance. *ATG3* demonstrated complete divergence between transcript and protein data, as its significant subtype-dependent mRNA differences were not mirrored at the protein level.

## 3. Discussion

Thymic epithelial tumors are rare neoplasms that exhibit molecular heterogeneity, as well as histological and clinical diversity, as they can be subdivided into morphological distinct types and accompanied by a wide range of paraneoplastic and autoimmune manifestations [1]. Although a variety of genetic modifications in molecules pathologically involved in oncogenesis pathways in TETs are known, the pathogenetic mechanisms, as well as predictive biomarkers, that could serve as potential therapeutic targets in TETs have not been fully clarified. Autophagy, a cellular process of product degradation and recycling with a dual role in both the induction and suppression of carcinogenesis in various cancer types, remains an uncharted pathogenetic mechanism in TETs [52]. The present study successfully highlighted for the first time the immunohistochemical expression of regulatory molecules involved in autophagy pathways (BECLIN, p62, LC3b, ATG3) in TETs.

In the present study, including 99 patients with TETs, immunohistochemical positivity of the examined autophagy pathway components (BECLIN, p62, LC3b, ATG3) was observed. BECLIN expression was detected in the vast majority of TETs, with a positivity rate of 87.7% and a predominantly strong staining intensity. BECLIN immunoexpression was associated with advanced Masaoka–Koga stage IV, as well as male gender. BECLIN is the major inductive autophagy regulator that interacts with DAPK (death-associated protein kinase), UVRAG (UV-radiation-resistance-associated gene protein), AMBRA1 (Activating Molecule in Beclin 1-Regulated Autophagy protein 1), and BIF1 (Bax interacting factor 1) molecules. This interaction results in the inhibition of binding of BECLIN with its inhibitor, BCL2 anti-apoptotic protein, and therefore in autophagy initiation. BECLIN has been shown to be involved in the carcinogenesis of various types of cancer [53,54,55].

High BECLIN expression has also been observed in various types of cancer and is often associated with advanced disease stages. Increased BECLIN expression has been reported in advanced triple-negative breast cancer [54], liver cancer [28], endometrioid ovarian carcinoma [54], gastric carcinomas [53,56], and colorectal carcinomas [53,54,55,56], as well as in patients with advanced melanoma [53]. Nevertheless, there are also several types of neoplasms, displaying decreased BECLIN expression [40,42,54,55,56,57], probably associated with the loss of BECLIN’s function as a tumor suppressor protein.

The significance of BECLIN in carcinogenesis is also evident from the fact that BECLIN induction contributes to proliferation and survival of cancer stem cells (CSCs) in chronic myeloid leukemia [41]. BECLIN activation also has been reported to trigger various cytokines. BECLIN for example promotes IL-6 (interleukin 6), which subsequently activates STAT3 (signal transducer and activator of transcription 3) factor, responsible for the survival and cellular proliferation of CSCs, resulting in infiltration and metastasis, as typically occurs in breast cancer [44]. BECLIN activation has been also correlated with HIF-1a (hypoxia inducible factor 1 subunit alpha), produced by the hypoxic environment of a tumor, through the activation of BNIP3 and NIX and the inhibition of BECLIN and BCL2 binding [54].

In the present study, immunohistochemical p62 expression was detected in only 17.7% of the examined cases, with the majority of cases displaying a mild staining intensity. Importantly, p62 immunoexpression was associated with aggressive types of TETs, specifically, B3 thymomas and TCs. p62 expression plays a significant pathogenetic role in many types of cancer and is associated with worse prognosis in patients with hepatocellular carcinoma [53,54,56] and nasopharyngeal carcinoma [53], as well as colon cancer [55]. The p62 molecule, acting as an autophagy cargo receptor (ACR), is responsible for the selective transport and engulfment of cytotoxic products for degradation in the autophagosome, playing an important part in late stages of the autophagy pathway [58,59]. The immunohistochemical p62 expression in TETs indicates p62/SQSTM1 accumulation in tumor cells and inhibition of p62 degradation. This finding could suggest autophagy suppression and subsequently promotion of cell proliferation, prevention of senescence and cell death, and progression of tumor growth [54,56]. This occurs through NRF2 (Nuclear factor erythroid 2-related factor 2), mTORC1, TRAF6 (TNF Receptor-Associated Factor 6), TNFa (Tumor Necrosis Factor a), and NFκΒ (Nuclear factor kappa-light-chain-enhancer of activated B cells) activation [38,39,43,53] and leads to ROS production and the induction of mitochondrial and DNA damage [54]. For example, according to Debnath et al., p62 accumulation caused by autophagy inhibition in breast cancer cells prevents degradation of the glycolysis mediator, PFKFB3 (6-phosphofructo-2-kinase/fructose-2,6-biphosphatase 3), further enhancing the Warburg effect and promoting tumor cell survival and proliferation, as well as metastasis [38]. Moreover, p62 accumulation leads to the inhibition of TWIST transcription factor degradation due to autophagy deficiency. As a result, TWIST activates the PI3K/AKT/mTOR pathway and contributes to the mesenchymal phenotype of tumor cells, resulting in EMT and metastasis [30,38,44].

In this study, cytoplasmic LC3b immunoreactivity was observed in only 14.1% of the examined patients with TETs, whereas almost all the investigated cases showed a moderate staining intensity. Cytoplasmic LC3b expression was mostly detected in TETs exhibiting favorable biological behavior (A, AB, B1, B2, MNT) compared to B3/TCs, a correlation that displayed, however, borderline statistical significance. LC3b protein participates in the elongation of the phagophore, as well as its transformation into a double-membraned ring and therefore the formation of the autophagosome [28,59]. LC3b functions as a receptor on the autophagosome membrane and is recognized by autophagy cargo receptors, such as p62 [28,59]. This study suggests a possible role of LC3b, which is involved in earlier stages of autophagy compared to p62, in the pathogenesis of non-B3/TCs.

Increased LC3b expression has been observed in a multitude of cancer types, specifically in hepatocellular carcinoma [32], glioblastoma [53], melanoma [32,53], triple-negative breast cancer [32,53,54,59], pancreatic cancer [55], and colorectal cancer [54,55]. In most of the studies, increased LC3b expression has been associated with advanced stages of the disease and poor prognosis. This is, however, not the first study to implicate an association between increased LC3b expression and favorable clinical behavior. There are neoplasms in which, due to the dual role of autophagy, low LC3 expression has been observed in advanced-stage cases such as ovarian cancer [54] and melanoma [53]. Similarly, according to Wu et al., in VHL (Von Hippel Lindau)-associated renal cell carcinoma, proteasomal degradation of LC3b is linked to autophagy inhibition and tumor progression [27].

This study further highlighted immunohistochemical cytoplasmic ATG3 expression with a positivity rate of 59.2% of the examined cases, mostly with an intense and a moderate staining intensity. There was no statistically significant correlation between ATG3 expression and WHO histological type or Masaoka–Koga stage. The ATG3 protein belongs to the panel of autophagy-related proteins (ATG), participating along with ATG7 and the ATG12-ATG5-ATG16L complex in the conjugation of PE to LC3a. This results in the conversion of the cytosolic form of LC3a, which is essential for elongation of the phagophore and autophagosome formation, into its lipidated form, LC3b [28]. Studies regarding the involvement of ATG3 in the carcinogenesis of specific cancer types are limited. Debnath et al. shows that ATG3 downregulation and, therefore, autophagy inhibition awakens tumor cells, resulting in metastatic niche formation [38], while Wu et al. report that ATG3 proteasome degradation stimulates metastasis in non-small cell lung cancer [27].

The combined transcriptomic and immunohistochemical analyses reveal a layered regulation of autophagy in TETs. *BECN1* showed a generally coherent gene–protein pattern, with higher mRNA levels in aggressive WHO subtypes and increased Beclin-1 protein in advanced Masaoka–Koga stages. Although no significant association with WHO histology was observed at the protein level, the overall trend suggests an increase in autophagy initiation in more progressive tumors. LC3B demonstrated a similar directional agreement, as both *MAP1LC3B* transcripts and LC3b protein were reduced in high-grade tumors, consistent with accelerated autophagic turnover limiting protein accumulation. In contrast, p62 exhibited only partial concordance. Although protein expression was enriched in B3 and TC tumors, *SQSTM1* mRNA levels were heterogeneous, suggesting strong modulation by autophagic flux. *SQSTM1* transcripts were substantially higher across TETs compared with the other autophagy-related genes, indicating a globally elevated baseline of p62 transcription. Within this overall pattern, the relatively increased mRNA levels observed in type A likely represent basal homeostatic transcription rather than true p62 accumulation, as protein expression in this subtype remains low. In contrast, the aggressive B3 and C tumors displayed pronounced p62 protein buildup, consistent with flux-dependent accumulation driven by both transcriptional activity and impaired autophagic processing. ATG3 showed clear divergence, with subtype-dependent mRNA differences not reflected at the protein level, implying post-translational regulation.

The rarity and heterogeneity of TETs, as well as limited scientific research data, concerning the association between the expression of molecules involved in autophagy pathway and TETs, make it difficult to draw reliable conclusions regarding both the pathogenetic and the prognostic role of autophagy-related proteins that could potentially serve as autophagy biomarkers in TETs. The paradoxical duality of autophagy and the fact that the inductive or suppressing role of autophagy in tumor growth is determined by different factors, such as tumor type, stage of neoplastic disease, genetic profile and tumor microenvironment, as well as the observation that autophagy prevents tumorigenesis in the early stages of a tumor but promotes cancer in the advanced stages of a tumor, make the discovery of autophagy biomarkers more complex, even in TETs [34,35]. Research on autophagy proteins has not yet clarified whether the pathogenesis of tumors, such as TETs, depends entirely on an autophagy mechanism or is simply due to an overaccumulation of autophagy factors, such as p62 and LC3 [38]. Similarly, it is not clear in a tumor, even in different WHO histological types of TETs, whether purely the early or late stages of autophagy are affected and to what extent selective autophagy, such as mitophagy, lipophagy, ferritinophagy, or pexophagy, is involved in tumorigenesis [57,59]. So far, it has been found that the only autophagy biomarkers that could be used are LC3 and p62. However, the stage of autophagy that biomarkers regulate in the pathogenesis of various cancer types, as well as the method for detecting post-translational modifications that autophagy proteins undergo, have not yet been elucidated, in order to be used as prognostic biomarkers [57]. Nevertheless, our study implicates BECLIN in advanced stages of TETs and p62, as an autophagy molecule involved in the late stages of the autophagy pathway in B3/TCs, while LC3b, as an autophagy-related protein, may rather participate in earlier stages of autophagy mechanisms in non-B3/TCs. The potential role of these molecules as autophagy biomarkers in patients with TETs for the evaluation of patients’ prognosis and TETs’ biological behavior requires further validation.

## 4. Materials and Methods

### 4.1. Study Population

This study analyzed archival formalin-fixed and paraffin-embedded (FFPE) tissue from 99 patients with TETs who underwent resection between 2009 and 2020 at Evangelismos General Hospital in Athens, Greece, with available medical records. Among the patients, 43 were men (43.4%) and 56 were women (56.6%), with a median diagnosis age of 62.5 years (ranging from 27 to 88). Tumors were classified according to the WHO system into various types: type A (12.1%), AB (22.2%), B1 (17.2%), B2 (19.2%), B3 (14.2%), micronodular thymoma with lymphoid stroma (MNT) (2%), and thymic carcinoma (13.1%) [60]. TET staging was based on the Masaoka–Koga staging system, in which tumors are categorized into four stages, according to the invasion into the thymic capsule, thymic surrounding tissue, and adjacent organs and the presence of locoregional metastases in pleura and pericardium or distant hematogenous or lymphogenous metastases [61]. Staging followed the Masaoka–Koga system, revealing that 19.1% were stage I, 38.2% stage IIa, 16.8% stage IIb, 19.1% stage III, 3.4% stage IVa, and 3.4% stage IVb. Positive surgical margins were noted in 29.2% of cases, and 59.7% of patients had coexisting thymoma-associated myasthenia gravis (TAMG). Treatment included chemotherapy for 28% and radiotherapy for 50% of patients, with six receiving both. Follow-up data were available for 37 patients, with a median duration of 37 months (ranging from 5 to 134 months). Patients and disease characteristics, as well as therapeutic modalities and outcomes, are shown in Table 1.

### 4.2. TMA Construction

One representative FFPE tissue block from each tumor was selected after reviewing all hematoxylin–eosin (H&E)-stained slides. Tissue micro arrays (TMAs) were then created using a manual tissue arrayer (TMA Model I, Beecher Instruments, Sun Prairie, WI, USA), with three to five 1.5 mm cores taken from each selected block and placed into positionally encoded arrays within eight recipient paraffin blocks. This approach included multiple cores from each case to account for histological tumor heterogeneity. Controls, including tonsils, placenta, and normal kidney tissue, were used during TMA construction.

### 4.3. Immunohistochemistry

Immunohistochemistry was performed on eight TMAs following established protocols. Sections were stained with primary antibodies targeting BECLIN (clone EPR20473, Abcam, Cambridge, UK; dilution 1:100), p62/SQSTM1 (clone EPR18351, Abcam, Cambridge, UK; dilution 1:2000), LC3b (clone EPR18709, Abcam, Cambridge, UK; dilution 0.1 µg/mL), and ATG3 (clone EPR4801, Abcam, Cambridge, UK; dilution 1:100–1:250).

Antigen retrieval was tailored for each primary antibody per the manufacturer’s guidelines. Heat-induced epitope retrieval (HIER) in Tris/EDTA buffer at pH 9.0 was applied for Beclin-1 and SQSTM1/p62, while citrate buffer at pH 6.0 was used for LC3B and ATG3.

The Envision (Dako, Agilent, Santa Clara, CA, USA) visualization system was used with DAB (3,3-diaminobenzidine) as a chromogen and hematoxylin as a counterstain. Manufacturer-recommended positive controls were included, and negative controls consisted of primary antibody omission or replacement with an irrelevant antiserum.

Immunohistochemical expression was assessed semi-quantitatively via the H-score, which multiplies staining intensity score (score 1 to 3) by the percentage of positive cells, yielding values between 0 and 300. Cytoplasmic staining was evaluated independently in the epithelial and lymphocytic components.

### 4.4. Statistical Analysis

Statistical analysis was conducted by an MSc biostatistician (G.L.) to assess the relationship between the immunohistochemical expression of BECLIN, p62, LC3b, and ATG3 and clinicopathological characteristics. Non-parametric tests, with corrections for multiple comparisons when necessary, were utilized for this examination. Survival analysis was carried out using Kaplan–Meier survival curves, with differences between the curves evaluated using the log-rank test. Numerical variables were categorized based on their median values, and a *p*-value of less than 0.05 was deemed statistically significant. The analysis was executed using the STATA 11.0/SE statistical software package version 11.0 for Windows.

### 4.5. UALCAN Analysis

UALCAN (The University of Alabama at Birmingham CANcer data analysis portal; https://ualcan.path.uab.edu/ (accessed on 20 January 2026)) is an interactive web resource designed for in-depth exploration of TCGA datasets [62]. In the present study, UALCAN was used to assess the transcriptomic expression of key autophagy-related genes across different histological subtypes of thymic epithelial tumors (TCGA-THYM cohort). Data were visualized as boxplots, and statistical comparisons between groups were performed automatically by the UALCAN platform using Student’s *t*-test, with *p* < 0.05 considered statistically significant. Moreover, overall survival (OS) analysis was conducted to evaluate the prognostic impact of gene expression levels. UALCAN stratifies patients into a high-expression group (top 25% of the cohort) and a medium/low-expression group (remaining 75%), enabling comparative survival assessment within the TCGA-THYM dataset.

## 5. Conclusions

In this study, we demonstrate for the first time the immunohistochemical expression of autophagy-related proteins (BECLIN, p62, LC3b, ATG3) in a large cohort of TETs, and their cytoplasmic expression was correlated with clinicopathological determinants, such as male gender, aggressive type of TETs, and advanced stage of disease. These results implicated a potential role of these molecules as prognostic biomarkers in TETs. Further research is needed in order to fully elucidate the uncharted molecular regulatory mechanisms of the dual and complex cellular process of autophagy in such a rare type of malignancy with clinicopathological and molecular heterogeneity and diversity.

## Figures and Tables

**Table 1:** Clinicopathological characteristics of 99 patients with TETs.

Parameter	Median	Range
**Age**	62.5 years	27–88 years
	**Number**	**%**
**Gender**		
Male	43/99	43.4%
Female	56/99	56.6%
**WHO subtypes**		
Type A	12/99	12.1%
Type AB	22/99	22.2%
Type B1	17/99	17.2%
Type B2	19/99	19.2%
Type B3	14/99	14.2%
Micronodular with lymphoid stroma (MNT)	2/99	2%
Thymic carcinoma (TC)	13/99	13.1%
**Masaoka–Koga stage**		
I	17/89	19.1%
IIa	34/89	38.2%
IIb	15/89	16.8%
III	17/89	19.1%
IVa	3/89	3.4%
IVb	3/89	3.4%
**Positive surgical margins**	14/48	29.2%
**Presence of myasthenia gravis**	34/57	59.7%
**Neoadjuvant chemotherapy**	11/39	28.2%
**Preoperative radiotherapy**	19/38	50%
Survival outcomes		
Alive, disease-free	25/37, follow-up 5–134 months	67.6%
Alive with disease	4/37, follow-up 28–65 months	10.8%
Dead of disease	8/37, follow-up 7–65 months	21.6%
**Presence of relapse**	4/35, follow-up 58–65 months	11.4%

**Table 2:** Expression of BECLIN, p62, LC3b, and ATG3 in TETs and correlations with clinicopathological parameters.

	Positivity Rate	H-Score, Median	H-Score, Range	Correlations with Clinicopathological Parameters
BECLIN high cytoplasmic expression	87.7%	87	0–100	Male gender
BECLIN high cytoplasmic expression		100	100–100	Masaoka–Koga stage IV
p62 high cytoplasmic expression	17.7%	0	0–20	Male gender
p62 high cytoplasmic expression		90	0–100	B3 thymomas, TC *
LC3b cytoplasmic expression	14.1%	None	None	Non-B3/TC * TETs
ATG3 cytoplasmic expression	59.2%	None	None	None

* Thymic carcinomas.

## Data Availability

The data presented in this study are available on request from the corresponding author. All transcriptomic datasets analyzed in this study are publicly accessible through the UALCAN (https://ualcan.path.uab.edu (accessed on 20 January 2026)) online platform. No additional datasets were generated by the authors.
